# Point Cloud Compression: Impact on Object Detection in Outdoor Contexts

**DOI:** 10.3390/s22155767

**Published:** 2022-08-02

**Authors:** Luís Garrote, João Perdiz, Luís A. da Silva Cruz, Urbano J. Nunes

**Affiliations:** 1 Department of Electrical and Computer Engineering, University of Coimbra, 3030-290 Coimbra, Portugal; garrote@isr.uc.pt (L.G.); joao.perdiz@isr.uc.pt (J.P.); lcruz@deec.uc.pt (L.A.d.S.C.); 2 Institute of Systems and Robotics, University of Coimbra, 3030-290 Coimbra, Portugal; 3 Instituto de Telecomunicações, University of Coimbra, 3030-290 Coimbra, Portugal

**Keywords:** point cloud, lossy compression, object detection, depth maps, depth filtering, machine learning

## Abstract

Increasing demand for more reliable and safe autonomous driving means that data involved in the various aspects of perception, such as object detection, will become more granular as the number and resolution of sensors progress. Using these data for on-the-fly object detection causes problems related to the computational complexity of onboard processing in autonomous vehicles, leading to a desire to offload computation to roadside infrastructure using vehicle-to-infrastructure communication links. The need to transmit sensor data also arises in the context of vehicle fleets exchanging sensor data, over vehicle-to-vehicle communication links. Some types of sensor data modalities, such as Light Detection and Ranging (LiDAR) point clouds, are so voluminous that their transmission is impractical without data compression. With most emerging autonomous driving implementations being anchored on point cloud data, we propose to evaluate the impact of point cloud compression on object detection. To that end, two different object detection architectures are evaluated using point clouds from the KITTI object dataset: raw point clouds and point clouds compressed with a state-of-the-art encoder and three different compression levels. The analysis is extended to the impact of compression on depth maps generated from images projected from the point clouds, with two conversion methods tested. Results show that low-to-medium levels of compression do not have a major impact on object detection performance, especially for larger objects. Results also show that the impact of point cloud compression is lower when detecting objects using depth maps, placing this particular method of point cloud data representation on a competitive footing compared to raw point cloud data.

## 1. Introduction

The increasing availability of ever more spatially detailed Light Detection and Ranging (LiDAR) data has both driven, and been the driver of, increasingly complex methods for the acquisition, processing, and subsequent information extraction from 3D point clouds. Due to their ability to represent virtually the physical world, they are especially useful in applications such as video gaming, virtual reality and robotic navigation, and within these the subset of autonomous driving applications poses specific challenges. However, the large amount of data generated when creating these representations causes problems in transmission, manipulation, and storage, especially in the scope of vehicular navigation and vehicle-to-vehicle or vehicle-to-infrastructure communication. Therefore it is necessary to reduce the amount of data transmitted between processing devices; a plausible solution might be attained by compressing the LiDAR point clouds.

The main purpose of this work is to analyze the impact of point cloud compression on object detection. Uncompressed point cloud data from the KITTI dataset as well as point clouds compressed at three compression ratios were used in the object detection study. Results were obtained using two different publicly available detection and classification architectures for point cloud data. Performance metrics were also computed for image-based object detection that uses depth data generated from the raw and compressed point clouds. Two common point cloud interpolation methods [1,2] were used with the YOLOv5m and YOLOv5l [3] image-based detection architectures.

Detection metrics obtained from using these architectures were used to analyze the robustness of the methods to lossy compression of the LiDAR data with three degrees of compression. Specifically, higher robustness in object detection was obtained on depth map-based data when using compressed point cloud data in the training set, compared to results obtained using other object detection methods. Object detection was affected by the compression of point cloud data in the testing set, with a degradation in the detection metrics when testing with lossily compressed point clouds. This empirical evaluation of the influence of lossy LiDAR point cloud compression on the performance of object detection methods is the main contribution of this work.

This paper is organized as follows. In Section 2, we briefly describe some background work relevant to the point cloud compression and projection and the depth-mapping methods used in the tests further ahead. Section 3 describes in more detail the compression algorithms, system architecture and object detection methods that were used, while the test results are shown in Section 4. Section 5 concludes the paper with remarks about the impact of compression on object detection from point clouds, with a final highlight on the pertinence of implementing and testing methods using intermediate representations (depth maps) for object detection.

## 2. Background

### 2.1. Point Cloud Compression

Over the years, several algorithms for point clouds and mesh compression have been proposed. Some of them operate at capture time in the sensor as described in [4] in the case of LiDAR point clouds, generally with low compression rates. The 3D Mesh method [5], which converts a 3D model into a set of polygons, is an extensively studied method, often used as a starting point for the development of others. Pauly and Gross [6] have proposed a method that converts a point cloud into a diversity of images—a height map—and, employing solely information on points and normals, discards vertex connection information. A self-similarity method developed by Hubo et al. [7] allows storage of additional point parameters by grouping them into clusters of surface patches. The Shape-Adaptive Wavelet Coding compression method introduced in [8] and the use of geometric primitives proposed by Schnabel et al. [9] show that high-quality renderings can be achieved through compression.

Real-time compression of point clouds has been achieved through voxelization and the incremental updating of 2D patches inside each voxel [10]. In [11], compression was attained through the use of Random Sample Consensus (RANSAC) to extract planes from a point cloud with the planes subsequently transformed into surfaces by Delaunay triangulation. There are also 2D representation methods, which alternatively propose mapping the point cloud into panoramic images [12] or, using additional laser scanner information, into 2D pixels [13]. A deep learning approach to compression can be seen in [14], where the authors propose employing a recurrent neural network to compress LiDAR data that had previously been converted to 2D images, while using a network with a residual block to decompress it.

Tree-based methods, in turn, make use of a tree structure to represent the point cloud. Octree structures for octant-based tree mapping of the point cloud were proposed by R. Schnabel et al. [15], J. Elseberg et al. [16], and Kammerl et al. [17]. In [15] prediction techniques were applied achieving high compression rates. In [16] an octree algorithm is proposed with high efficiency in storage and compression of a large volume of 3D data, without any loss of precision. In [17] a real-time compression algorithm is proposed to perform spatial decomposition based on octree data structures. This method also encodes point attributes using a range coder and removes temporal redundancy in dynamic point clouds. Hubo et al. [18] used kd-trees, reducing the memory footprint and preserving performance, while Gumhold et al. [19] used prediction trees that improved compression rates in bits per point. The compression method implemented in [20] uses quad-trees and binary tree partitions to reduce the complexity of the bounding boxes relative to those that would be created by using solely octree partition, thus saving memory. Recently, to address requests from the industry sector, the MPEG standardization group developed two 3D point cloud coding algorithms that are at the moment the top-performing solutions for compression of 3D point clouds [21,22]. One of them, Geometry-based Point Cloud Compression (G-PCC), is geared towards the efficient compression of sparse and very sparse point clouds like those typical of LiDAR. G-PCC is based on octree decompositions of the point cloud geometrical information and the transform coding of the point-attribute information. The other point cloud encoder proposed by MPEG, Video-based Point Cloud Compression (V-PCC), is a better fit for compressing dense point clouds but poorer for sparser ones. V-PCC follows a much more complex coding paradigm involving the projection of the 3D points onto 2D planes to form 2D images representing both the geometry (depth) information and attribute information. These 2D images are then encoded using legacy video encoders like High Efficiency Video Coding (HEVC/H.265) [23] or VersatileVideo Coding (VVC/H.266) [24], which achieve very high coding efficiency when applied to dense point clouds. Both of these two encoders have been designed to achieve high efficiency point cloud encoding while ensuring low processing complexity and ease of hardware implementation. Due to the very good performance reported when encoding sparse point clouds and very low complexity and small memory footprint, the G-PCC encoder was chosen to compress the LiDAR data used in this research study.

### 2.2. Depth-Mapping Point Clouds into 2D Images

Some methods make use of point cloud data indirectly, not for representation in 3D space but through conversion into depth information, which can then be mapped into a 2D image to be used for object detection and classification. In [1] several up-sampling methods for such a purpose are presented and evaluated, and compared with the novel approach proposed in [1], which consists of a modified bilateral filter that has a weighing function dependent on the range dispersion found in the mask, such that points in the same plane can be clustered together. The method obtained good edge preservation between surfaces in different planes when projecting a point cloud and its depth information onto a 2D image, needing no more data than that provided by a single-point LiDAR.

## 3. Methods

### 3.1. Point Cloud Compression

The LiDAR data used in this work, represented as 3D point clouds, were compressed using the MPEG G-PCC coder in octree geometry-only intracoding mode. While G-PCC supports a second geometry encoding method, *trisoup*, some preliminary tests done using LiDAR point clouds showed that the encoding performance of *trisoup* mode on those test point clouds was lower than that of *octree* mode, perhaps due to the high sparsity of that data. Since the *trisoup* mode is also more complex than the *octree* mode it was decided to use the latter. The implementation used was MPEG’s TMC13 version 11 [25], and three quality/compression operating points were chosen to produce three levels of fidelity of the decompressed LiDAR geometry information. Since G-PCC can compress only point clouds with coordinates expressed in a regular voxelized grid, prior to compression the point clouds were voxelized to a discrete grid with 20-bit precision in each coordinate. While it is unusual to use voxelization depths so large (more common values range from 10 to 12 bits) it was decided to use 20-bit precision to make sure that any loss in the representation of the LiDAR point clouds was due only to the compression process, not to the voxelization. After decompression/reconstruction, all point clouds were converted back to the original coordinate system. The compression ratio—hence the quality of the decoded point cloud—was controlled through adjustment of the MPEG G-PCC *positionQuantizationScale* parameter where the three values chosen were 1/1024, 1/512 and 1/256 corresponding to high-, medium- and low-compression degrees, henceforth represented by G3, G2 and G1. It was verified empirically that varying other encoder parameters had no significant effect on the encoding performance of G-PCC when applied to the LiDAR point clouds selected for use in this empirical study. The G-PCC configuration parameters used for each of the three coding qualities are listed in Table 1, where *positionQuantizationScale* controls the compression degree and *disableAttributeCoding=0* enforces encoding of only the geometry information of the point cloud. These three quality settings resulted in LiDAR point clouds compressed with bitrates, compression factors and associated compression-related distortions as listed in Table 2.

Following common practice, the bitrates are expressed as the ratio of the number of bits in the compressed representation (bitstream) to the number of points in the input point cloud. The compression factors reported are the ratio of the number of bits per point before compression i.e., 192 bits for the case of 3 coordinates per point and 64 bits per coordinate, to the number of bits per point after compression (second column of Table 2). The listed D1 Peak Signal to Noise Ratio (D1 PSNR) distortion values measure the symmetrical distance between the uncompressed and reconstructed point cloud (after compression) computed as the logarithm of the inverse of the squared minimum distance between points of both point clouds, as defined e.g., in Annex C of [26] or in [27].

**Figure 1:**
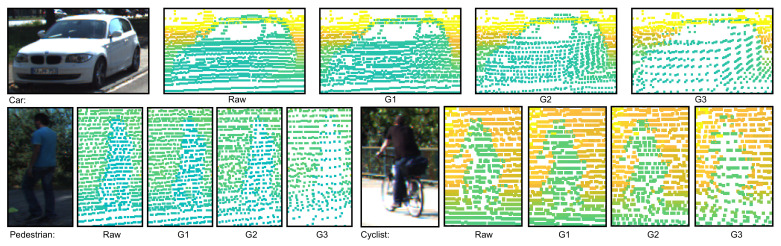
Examples of point cloud representations for each of the three classes of objects in the KITTI dataset. From left to right in each image we can see the RGB image, a representation of the raw point cloud, and point cloud representations at compression degrees G1, G2 and G3.

Examples of the effect of compression on point clouds representing objects from different classes can be seen in Figure 1. In this and subsequent images, a colored representation of either point clouds or depth maps was used in which objects or planes closer to the sensor are colored in blue tones, with tones warming to green and then yellow for objects and planes further away from the sensor.

### 3.2. Object Detection Methods

#### 3.2.1. Complex-YOLO

The Complex-YOLO [28] method is an expansion of the YOLOv2 method, a fast 2D object detector for RGB images. It converts the point cloud into a Bird’s-Eye-View RGB-map and, using an angular regression strategy and an Euler-Region Proposal Network (E-RPN), estimates and localizes 3D multiclass bounding boxes.

#### 3.2.2. PointNet++

PointNet [29] is one of the pioneer methods that operates directly on a set of points, learning a spatial coding for each one and clustering all individual point features to a global point cloud signature. PointNet++ presents an architecture for point set segmentation and classification that consists of a hierarchical neural network using PointNet recursively in small point sets. By exploiting metric space distances, local features are extracted from small neighborhoods and such features are further grouped in larger point sets until the features of the whole point set are reached. With those final features, segmentation and classification are then done.

#### 3.2.3. Point Cloud to Image

**Figure 2:**
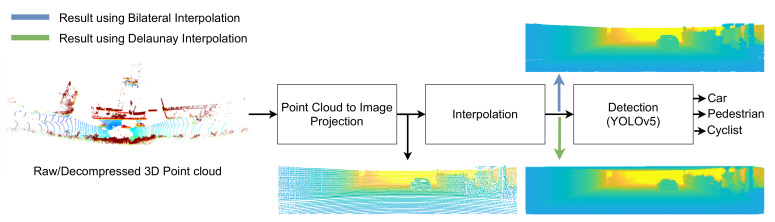
Pipeline of the object detection process for images projected from input 3D point clouds (raw or decompressed). Interpolation can be carried out either with Delaunay triangulation or the customized bilateral filter—the grayscale images show an example of that interpolation for either process.

The use of point cloud to image conversion in object detection was explored in [1,2]. In our work, the object detection pipeline illustrated in Figure 2 was deployed. Based on the aforementioned works, it used as input an image obtained after applying a custom bilateral filter interpolation [1] or an image obtained after applying Delaunay triangulation.

**Figure 3:**
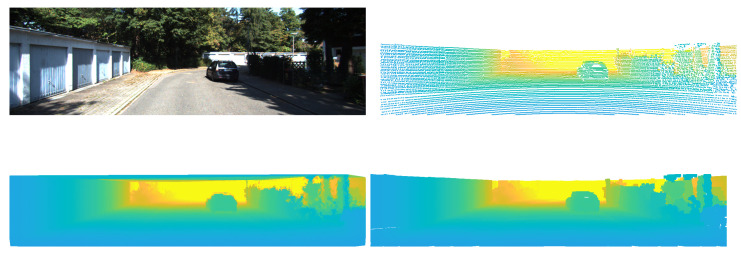
An example of an image from KITTI and its corresponding representations. Clockwise from top left: the RGB frame, its corresponding point cloud, the grayscale image obtained from the point cloud using a bilateral filtering method, and the grayscale image obtained from the point cloud using Delaunay triangulation.

**Figure 4:**
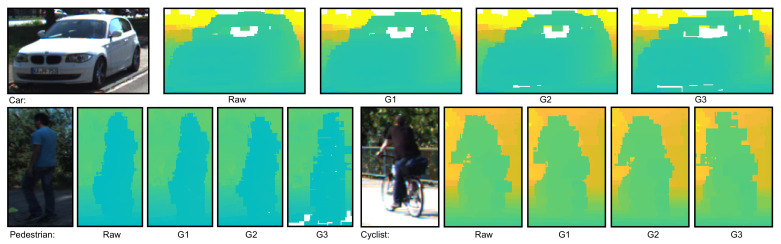
An example of the object classes that were considered from KITTI, with the depth images generated using the bilateral filtering method from point clouds compressed at different degrees. For each object class, from left to right: RGB image, depth images computed from data at G1, G2 and G3 compression degrees.

**Figure 5:**
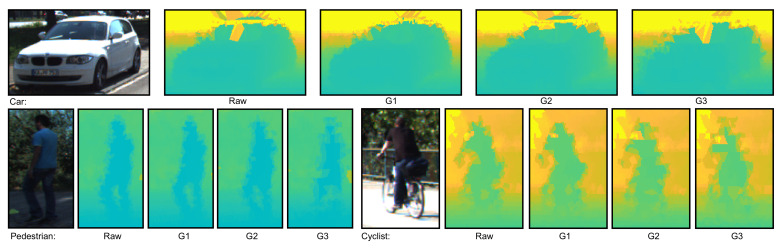
An example of the object classes that were considered from KITTI, with the depth images generated using the Delaunay Triangulation interpolation method from point clouds compressed at different degrees. For each object class, from left to right: RGB image, depth images computed from data at G1, G2 and G3 compression degrees.

For object detection, the YOLOv5 architecture family [3] was used. Both interpolation methods allow up-sampling of the sparse representation obtained after projecting the point cloud points into the image frame (see Figure 3). The bilateral filter uses a sliding window mask and clustering strategy. The depth values found in the mask are clustered according to their belonging to a foreground or background plane in the mask. Finally, a weighting function was used to compute the new depth value of the mask’s central point, considering foreground and background categorization and distance of the points. The Delaunay triangulation interpolation computed the depth values using the triangle mesh obtained after it was appplied to all non-empty pixels in the image. Each pixel’s depth value corresponds to the depth obtained for the pixel’s coordinates in an overlapping triangle. Figure 4 shows examples of how objects in KITTI are represented as depth maps using the bilateral filter method for interpolation, while Figure 5 shows the same examples after transformation with Delaunay triangulation.

## 4. Experimental Validation

To test the adequacy of the applied data compression methods, we decided to compare them using the well-known KITTI dataset [30], using 70% of the *KITTI training dataset* for training the aforementioned methods. The remaining 30% of the *KITTI training dataset* were compressed for use in the evaluation of the trained models. The encoder and compression degrees are those described in Section 3.1.

Two distinct and publicly available architectures for point cloud-based object detection were used in this study. Compression adequacy was also tested when using the point clouds as source data for images used to train image-based object detection architectures; for this case we employed YOLOv5m and YOLOv5l. Within the scope of this study, the following training and testing of object detection models were carried out:

- Training a Complex-YOLO object detection architecture model using raw data and testing it using raw and decompressed point cloud data. Precision, Recall, F1-score and Intersection over Union (IoU) metrics were used to evaluate model performance.
- Training a PointNet++ model using raw data and test it using raw and decompressed point cloud data. IoU is used to evaluate model performance.
- Training YOLOv5l and YOLOv5m models with images converted from raw point cloud data using bilateral filter interpolation, and testing them in images created from either raw or decompressed point cloud data, using the same interpolation method as that in training. Precision, Recall and mean Average Precision (mAP) metrics were used to evaluate model performance.
- Training YOLOv5l and YOLOv5m models with images converted from raw point cloud data using Delaunay triangulation interpolation, and testing them in images created from either raw or decompressed point cloud data, using the same interpolation method as that in training. Precision, Recall and mAP metrics were used to evaluate model performance.
- Results were also obtained for object detection using training and testing sets prepared with different methods. A training model was obtained with YOLOv5m and Bilateral Interpolation, while images created with Delaunay triangulation on G2 decompressed point cloud data were used for the testing set. Precision, Recall and mAP metrics were used to evaluate model performance.

### 4.1. Point Cloud-Based Architectures

For the Complex-YOLO architecture we trained the weights using raw data for 100 epochs. Weights that scored the highest average precision (on intermediate evaluations) were obtained on epoch 82; these were subsequently used to evaluate detection metrics for both raw and decompressed data. We also evaluated raw and decompressed data using the weights from a 300-epoch training run; these produced consistently lower metrics than those presented here.

Table 3 shows results for the evaluation of raw and decompressed point cloud data. Results for raw data show high performance when averaging (weighted) across all classes.

All compression levels were evaluated using weights models trained with raw point clouds. The results were markedly inferior to those obtained using raw point clouds, with major drops in precision when detecting non-car objects. It could be seen that non-car classes were responsible for most of the drop in the average precision metric, as well as in the other metrics shown. The effect of compression on the average IoU, which decreased from 0.7621 to 0.5497, was smaller than the effect on detection precision, which decreased from 0.88 to 0.34.

**Figure 6:**
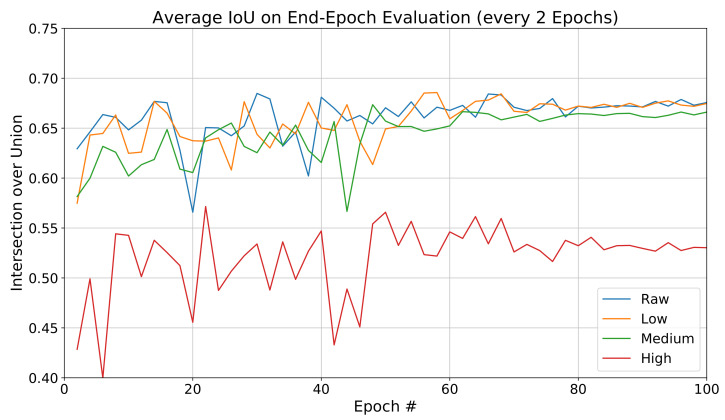
PointNet++ IoU performance evolution for each degree of compression (model trained with raw data). Test data is evaluated every two epochs.

For the PointNet++ architecture we obtained the final and intermediate IoU values for the training sessions. IoU average values for raw and decompressed point clouds are summarized in Table 4, and the evolution of IoU during training is shown in Figure 6. Network evaluations were made every two epochs, with different points of distinct degrees of data compression converge. Two out of the three compression levels attained similar IoU values to those of raw data, with a significant penalty observed for IoUs of highly compressed data.

### 4.2. Point Cloud to Image Architectures

#### 4.2.1. Point Cloud Image Conversion

To validate the architecture depicted in Figure 2 with the KITTI dataset, the label format and dataset structure were modified. In particular, the point cloud label format was converted from the KITTI format into the PASCAL Visual Object Classes(PASCAL VOC) using a publicly available library vod-converter, found at https://github.com/umautobots/vod-converter (accessed on 5 January 2022). This allowed us to convert the bounding boxes and labelling from KITTI directly into a format readily usable in YOLOv5. Using this format allows us to test the detection suitability of the multiple network architectures associated with YOLO, which have vastly different depths and, consequently, very different parameter counts. We excluded the largest networks and focused our analysis on the YOLOv5l and YOLOv5m sub-architectures.

Object detection models were evaluated for different image sets obtained from raw or differently compressed point cloud datasets, with the same training-test split as that used for Complex-YOLOv3 and PointNet++. Results from images created using bilateral filter interpolation are shown in Table 5 for YOLOv5l and Table 6 for YOLOv5m. For all image sets, performance assessments were always performed with the best weights network obtained when training the model with images from raw data.

A small degradation in object detection accuracy was observed from G1 to G2 levels, with a much larger gap for G3 compression. Results for low and medium compression are roughly equivalent to those obtained from the evaluation of directly-converted point cloud data, which shows the adequacy of the Bilateral Filter approach when dealing with point clouds in differing states of compression.

As an extra step, we evaluated images created using Delaunay triangulation with the network trained using images from the bilateral filter. The results are shown in Table 7. The detection metrics can be considered reasonable, given that the training and evaluation datasets were created from the same dataset using two different methods.

#### 4.2.2. Detection Using Images Created with Delaunay Triangulation

To compare results obtained using the custom bilateral filter described in [1] with another point cloud up-sampling method, we used images created using Delaunay triangulation in the same manner as described in [1], and tested as in [1,2] on the KITTI dataset. Delaunay triangulation is employed to “fill-in” a 2D representation of the point-cloud with data interpolated from sparse Depth Map data, allowing a depth-based image to be used in place of either the standard KITTI images or point clouds even though it is based only on the latter. Thus the labelling conversion is the same as that used in the previous analysis. Detection results for these images using either the YOLOv5l or YOLOv5m sub-architectures are shown in Table 8 and Table 9, respectively.

### 4.3. Discussion

From the results summarized in Table 3, Table 4, Table 5, Table 6, Table 7, Table 8 and Table 9, the detection performance obtained when using decompressed point clouds was close to the level obtained using raw point clouds. The results showed decreasing performance with increasing compression degree. Due to the smaller size of the pedestrian and cyclist objects, the distortion effects due to the higher levels of compression led to “noisier” representations of those types of objects, resulting in lower detection performance. Moreover the performance decrease was higher for higher compression levels. For the car objects, due to their larger sizes, the image interpolation still correctly represented the object, with smaller drops in performance.

For 3D-based object detection methods, a steady decrease in performance was seen with each step of increased compression degree. The PointNet++ architecture is robust to a certain degree to different levels of lossy compression, although it showed poorer performance for object detection from point cloud data with a higher degree of compression. It appeared to be, however, more stable than Complex-YOLO over different degrees of compression, which may have been due to its approach of using local point sets. For Complex-YOLO we saw a steady decline in performance when going from raw data to higher degrees of compression; this empirical evidence was made clearer by the larger set of metrics available in the ComplexYOLO architecture compared to PointNet++. The observed trend for the interpolation-based methods, with smaller objects suffering the highest penalties in detection metrics for higher compression degrees, was also verified in the case of 3D-based object detection.

## 5. Conclusions

While it can be said that the different metrics computed for each employed architecture made direct comparisons difficult, it was noticeable that high rates of compression had, in general, a significantly detrimental effect on object detection performance, especially when discussing modalities operating directly on 3D data.

The results also showed that the use of point cloud-to-depth map conversion was more robust to the changes introduced by the compression with different degrees of information loss. This robustness could be attributed in part to the interpolation strategies employed, which up-sample points and in some cases soften the influence of outliers by considering multiple points in a mask.

Furthermore, object detection of smaller objects is shown to be consistently more affected by point cloud data compression.

In conclusion, the PointNet++ and the presented point cloud-to-image object detection pipeline are robust to a medium degree of lossy compression, with only a small reduction in performance, and can be considered in application scenarios where only lossy point clouds are available. Considering the compression factors that can be achieved as shown in Table 2, and the fact that these will allow vehicle-to-vehicle communication to proceed with much lower data transmission rates than would otherwise be necessary, the small degradation in object detection might, in some circumstances, be considered an acceptable trade-off. Future work on this topic can include the modification of object detection methods by introducing preprocessing stages that include local interpolation strategies.

## Figures and Tables

**Table 1:** MPEG G-PCC coder parameters used for qualities G1, G2 and G3.

G-PCC Parameters	Quality		
	G1	G2	G3
mode	0	0	0
mergeDuplicatedPoints	1	1	1
neighbourAvailBoundaryLog2	8	8	8
intra_pred_max_node_size_log2	6	6	6
positionQuantizationScale	1/256	1/512	1/1024
disableAttributeCoding	1	1	1

**Table 2:** Average bitrates, compression factors and point-to-point distances (D1 PSNR) of compressed point clouds.

Quality	Bits per Point (bpip)	Compression Factor	D1 PSNR (dB)
G1	4.859	39.51	83.04
G2	2.775	69.19	77.02
G3	1.373	139.84	71.00

**Table 3:** Complex-YOLO architecture performance evaluated in raw and decompressed point clouds.

Class	Precision				Recall				F1-Score				IoU			
	Raw	G1	G2	G3	Raw	G1	G2	G3	Raw	G1	G2	G3	Raw	G1	G2	G3
Car	0.97	0.98	0.88	0.65	0.98	0.97	0.91	0.68	0.95	0.82	0.81	0.71	-	-	-	-
Pedestrian	0.77	0.56	0.36	0.10	0.92	0.78	0.51	0.15	0.75	0.51	0.47	0.23	-	-	-	-
Cyclist	0.91	0.53	0.42	0.26	0.95	0.66	0.52	0.35	0.88	0.51	0.47	0.38	-	-	-	-
Average	0.88	0.68	0.55	0.34	0.95	0.81	0.65	0.39	0.86	0.61	0.59	0.44	0.7621	0.6634	0.6317	0.5497

**Table 4:** Average IoU performance of PointNet++ model across classes, for varying degrees of point cloud compression.

IoU Average (Epoch 100)			
Raw	G1	G2	G3
0.6756	0.6747	0.6661	0.5303

**Table 5:** Evaluation metrics for KITTI point cloud data compressed at different degrees, converted with the Bilateral Filter method and evaluated using the best YOLOv5l model. The resulting model, with the best weights, was obtained over the course of 200 epochs using a batch size of 10.

Class	Precision				Recall				mAP			
	Raw	G1	G2	G3	Raw	G1	G2	G3	Raw	G1	G2	G3
Car	0.917	0.900	0.879	0.867	0.869	0.881	0.880	0.816	0.938	0.938	0.932	0.882
Pedestrian	0.937	0.926	0.886	0.867	0.724	0.733	0.730	0.599	0.837	0.833	0.821	0.705
Cyclist	0.893	0.926	0.845	0.764	0.682	0.694	0.680	0.416	0.789	0.782	0.768	0.499
Average	0.915	0.899	0.870	0.833	0.758	0.770	0.763	0.610	0.855	0.851	0.840	0.695

**Table 6:** Evaluation metrics for KITTI point cloud data compressed at different degrees, converted with the Bilateral Filter method and evaluated using the best YOLOv5m model. The resulting model, with the best weights, was obtained over the course of 200 epochs, with a batch size of 20.

Class	Precision				Recall				mAP			
	Raw	G1	G2	G3	Raw	G1	G2	G3	Raw	G1	G2	G3
Car	0.888	0.849	0.898	0.814	0.848	0.865	0.827	0.788	0.917	0.916	0.908	0.850
Pedestrian	0.927	0.879	0.932	0.675	0.665	0.706	0.665	0.605	0.800	0.799	0.773	0.632
Cyclist	0.848	0.808	0.869	0.732	0.674	0.680	0.614	0.380	0.762	0.754	0.735	0.456
Average	0.887	0.875	0.899	0.740	0.729	0.750	0.702	0.591	0.827	0.823	0.806	0.646

**Table 7:** Evaluation metrics for Medium (G2) compression KITTI point clouds converted with the Delaunay triangulation method and evaluated using the best YOLOv5m model obtained with Bilateral Filter-generated images.

Class	Precision	Recall	mAP
Car	0.714	0.682	0.738
Pedestrian	0.779	0.420	0.522
Cyclist	0.707	0.559	0.608
Average	0.733	0.554	0.623

**Table 8:** Evaluation metrics for KITTI point cloud data compressed at different degrees, from raw to high, converted with the Delaunay Triangulation and evaluated using the best YOLOv5l model, trained over the course of 200 epochs with a batch size of 10.

Class	Precision				Recall				mAP			
	Raw	G1	G2	G3	Raw	G1	G2	G3	Raw	G1	G2	G3
Car	0.922	0.887	0.877	0.853	0.854	0.876	0.827	0.774	0.935	0.934	0.906	0.864
Pedestrian	0.958	0.930	0.901	0.672	0.724	0.749	0.693	0.636	0.829	0.815	0.778	0.665
Cyclist	0.896	0.865	0.866	0.832	0.701	0.727	0.692	0.516	0.804	0.799	0.781	0.648
Average	0.925	0.894	0.881	0.785	0.760	0.784	0.737	0.642	0.856	0.849	0.822	0.725

**Table 9:** Evaluation metrics for KITTI point cloud data compressed at different degrees, converted with the Delaunay Triangulation and evaluated using the best YOLOv5m model, trained over the course of 200 epochs with a batch size of 20.

Class	Precision				Recall				mAP			
	Raw	G1	G2	G3	Raw	G1	G2	G3	Raw	G1	G2	G3
Car	0.855	0.879	0.882	0.842	0.859	0.848	0.796	0.759	0.917	0.916	0.888	0.849
Pedestrian	0.893	0.922	0.894	0.594	0.718	0.688	0.658	0.617	0.791	0.789	0.747	0.605
Cyclist	0.842	0.847	0.857	0.805	0.713	0.693	0.634	0.468	0.779	0.774	0.748	0.604
Average	0.863	0.883	0.878	0.747	0.763	0.743	0.696	0.614	0.829	0.827	0.794	0.686
